# Analyses of Potential Predictive Markers and Survival Data for a Response to Sunitinib in Patients with Metastatic Renal Cell Carcinoma

**DOI:** 10.1371/journal.pone.0076386

**Published:** 2013-09-27

**Authors:** Juana Dornbusch, Aristeidis Zacharis, Matthias Meinhardt, Kati Erdmann, Ingmar Wolff, Michael Froehner, Manfred P. Wirth, Stefan Zastrow, Susanne Fuessel

**Affiliations:** 1 Department of Urology, Dresden University of Technology, Dresden, Germany; 2 Institute of Pathology, Dresden University of Technology, Dresden, Germany; Maastricht University Medical Center, The Netherlands

## Abstract

**Background:**

Patients with metastatic clear cell renal cell carcinoma (ccRCC) are frequently treated with tyrosine kinase inhibitors (TKI) such as sunitinib. It inhibits angiogenic pathways by mainly targeting the receptors of VEGF and PDGF. In ccRCC, angiogenesis is characterized by the inactivation of the *von Hippel-Lindau* gene (VHL) which in turn leads to the induction of HIF1α target genes such as CA9 and VEGF. Furthermore, the angiogenic phenotype of ccRCC is also reflected by endothelial markers (CD31, CD34) or other tumor-promoting factors like Ki67 or survivin.

**Methods:**

Tissue microarrays from primary tumor specimens of 42 patients with metastatic ccRCC under sunitinib therapy were immunohistochemically stained for selected markers related to angiogenesis. The prognostic and predictive potential of theses markers was assessed on the basis of the objective response rate which was evaluated according to the RECIST criteria after 3, 6, 9 months and after last report (12–54 months) of sunitinib treatment. Additionally, VHL copy number and mutation analyses were performed on DNA from cryo-preserved tumor tissues of 20 ccRCC patients.

**Results:**

Immunostaining of HIF-1α, CA9, Ki67, CD31, pVEGFR1, VEGFR1 and -2, pPDGFRα and -β was significantly associated with the sunitinib response after 6 and 9 months as well as last report under therapy. Furthermore, HIF-1α, CA9, CD34, VEGFR1 and -3 and PDGRFα showed significant associations with progression-free survival (PFS) and overall survival (OS). In multivariate Cox proportional hazards regression analyses high CA9 membrane staining and a response after 9 months were independent prognostic factors for longer OS. Frequently observed copy number loss and mutation of VHL gene lead to altered expression of VHL, HIF-1α, CA9, and VEGF.

**Conclusions:**

Immunoexpression of HIF-1α, CA9, Ki67, CD31, pVEGFR1, VEGFR1 and -2, pPDGFRα and -β in the primary tumors of metastatic ccRCC patients might support the prediction of a good response to sunitinib treatment.

## Introduction

Metastatic clear cell renal cell carcinoma (ccRCC) is an incurable malignancy due to resistance to chemotherapy and in 80–95% of the cases to immunotherapy [1], [2]. The treatment perspectives and prognosis of patients with metastatic ccRCC were significantly improved by the understanding of the molecular pathogenesis of this tumor entity which led to the development of targeted therapeutics such as tyrosine kinase inhibitors (TKI). The TKI sunitinib (sunitinib malate; Sutent®) targets amongst others the receptors of vascular endothelial growth factor (VEGF) and platelet-derived growth factor (PDGF). It is approved worldwide for first-line treatment of advanced metastatic ccRCC and significant objective response rates of up to 47% have been reported [3], [4]. Nevertheless, a number of patients with metastatic ccRCC exhibited no clinical benefits from sunitinib treatment [5]. The identification of prognostic and predictive markers that are associated with a longer progression-free survival and a sunitinib-response, respectively, is required to enhance outcome of patients with advanced RCC by specific therapies.

Previous studies suggested a relationship between inactivation of the *von Hippel-Lindau* gene (VHL) by mutations, copy number losses and/or promoter methylation and the development of metastatic ccRCC as well as a poor outcome of the patients [6], [7], [8], [9]. The protein encoded by the VHL gene is a tumor-suppressor and part of an E3 ubiquitin ligase complex that targets the hypoxia-inducible factor 1α (HIF-1α) for ubiquitination and proteasomal degradation [10]. Beside the regulation of HIF-1α and the resulting influence on angiogenesis, cellular metabolism and cell growth, VHL is involved in many cellular processes including cell cycle regulation, extracellular matrix assembly, cytoskeleton stability and apoptosis [11]. Angiogenesis is essential for tumor growth and metastasis, thus VEGF, the most potent mediator of vessel formation [12], is the final target of TKIs which are used for treatment of ccRCC such as sunitinib, sorafenib, axitinib and pazopanib. However, there is currently a lack of prognostic and predictive biomarkers for response to TKI treatment. Recent data delineated an association of low carbonic anhydrase IX (CA9) levels and poor survival of patients with metastatic ccRCC and lower response rates to TKI treatment [13], [14]. The tumor expression levels of VHL, the endothelial marker CD31, PDGFRα, VEGF and the inhibitor of apoptosis survivin (SVV) are supposed to be important markers for prognosis and outcome of patients with advanced RCC [15], [16], [17], [18], [19]. The applicability of such molecular markers for prediction of a sunitinib response was demonstrated by recent studies. For example, overexpression of HIF-1α and a strong expression of VEGFR2 were associated with higher response rates to sunitinib [20], [21]. Furthermore, adverse effects like hypertension (HTN) and the hand-foot syndrome (HFS) appear to be associated with a better response to sunitinib and longer overall survival (OS) [22], [23].

In addition to known angiogenic factors (VEGFA and its upstream regulators) and their corresponding receptors (VEGFRs and PDGFRs) further biomarkers, which are directly or indirectly involved in the angiogenesis signalling network, were selected for the systematic investigation of their usability for response prediction. NRP-1 is a co-receptor for VEGFA that can enhance the VEGFR2 mediated angiogenic signals of VEGFA [24]. An elevated NRP-1 protein expression has been associated with a worse prognosis in several tumor entities [25]. VEGFA_165_B is a recently identified anti-angiogenic isoform of VEGFA and the balance of VEGFA_165_B to total VEGFA may have implications for therapy [26]. The microvessel density (MVD) can be assessed by CD31 and CD34 staining and gives important information on tumor vascularization which might be important for a response to TKI treatment [27], [28]. Also for SVV a functional relationship with induction of angiogenesis was reported for several tumor entities [29], [30]. Ki67 was used as reference marker due to its well-known prognostic relevance for ccRCC [30], [31]. Therefore, this study was aimed at the evaluation of the VHL status (mutation and copy number alterations), tumor protein expression of angiogenesis-associated genes (VHL, HIF-1α, CA9, CD31, CD34, VEGFA, VEGFA_165_B, NRP-1, VEGFR1, -2 and 3, pVEGFR1 and -2, PDGFRα and -β, pPDGFRα and -β), known prognostic markers (Ki67 and SVV) and treatment associated adverse effects with regard to prediction of the response to sunitinib treatment. The analyzed molecular markers were compared with established clinical prognostic factors in ccRCC such as T stage (pT), Fuhrman grade (G), primary lymph node status and distant metastasis (combined M/N stage) as well as TNM staging. Our investigations have shown that the immunoexpression of HIF-1α, CA9, Ki67, CD31, pVEGFR1, VEGFR1 and -2, pPDGFRα and -β in the primary tumors of metastatic ccRCC patients might support the prediction of a good response to sunitinib treatment.

## Materials and Methods

### Patients

The study was approved by the institutional review board of the Medical Faculty at the Technical University of Dresden (EK59032007 and EK195092004). Written informed consent was obtained from each patient. In the present study a cohort of 42 sunitinib-treated patients with histologically proven ccRCC were selected for different analyses. Tumor nephrectomies were conducted between 1997 and 2010, following diagnosis of metastases (at time of tumor diagnosis or later) patients started a TKI therapy with sunitinib. A total of 11 patients were previously treated with a cytokine immunotherapy (interleukin-2 or interferon α) and chemotherapy (5-Fluorouracil) whereas 4 patients received sorafenib before sunitinib treatment. The patient characteristics are summarized in Tables 1 and 2. Sunitinib was self-administered orally at a daily dose of 50 mg daily in repeated 6 week cycles of 4 weeks on treatment followed by a 2 weeks off schedule. Dose reductions to 37.5 mg and 25 mg sunitinib were allowed on the basis of individual tolerability. Computed tomography (CT) scans were obtained before treatment start and after every 2 cycles (3 months) of therapy. The objective response rate was evaluated according to the Response Evaluation Criteria in Solid Tumors (RECIST) version 1.0 [32]. Partial response and stable disease were summarized as objective response rate. Patients with mixed response, e.g. having some lesions reduced in size under therapy and in parallel developing a new small lesion, further received sunitinib therapy and were therefore also considered as responders. Due to the restricted number of possible further therapy options at this time the clinical decision was made more restrained in such cases. Complete response was not achieved in any of the patients. Patients with progression were considered as non-responders. Adverse effects of sunitinib such as HFS and HTN were also registered according to current guidelines.

**Table 1:** Characteristics of patients.

Parameter	Case number
**Total patients**	42 (100%)
**Sex**	
Male	29 (69%)
Female	13 (31%)
**Age at surgery**	
Median	64
Interquartile range (IQR)	56–70
**Age at initiation of therapy**	
Median	67
IQR	59–70
**Tumor size in cm (n** = **34)**	
Median	7.5
IQR	5.4–11.3
**pT**	
1	10 (23.8%)
2	3 (7.1%)
3	27 (64.3%)
4	2 (4.8%)
**Fuhrman grading**	
1	1 (2.4%)
2	20 (47.6%)
3	12 (28.6%)
4	9 (21.4%)
**Primary lymph node and distant metastasis**	
Yes (M1/N+)	28 (66.7%)
No (M0/N0)	14 (33.3%)
**TNM stage**	
I	4 (4.8%)
II	2 (4.8%)
III	11 (26.2%)
IV	25 (59.5%)

**Table 2:** Follow up data of patients.

Parameter	Months
**Median follow up (months)** 1	
All patients (n = 42)	
Median	39.6
IQR	13–45
Deceased patients (n = 28)	
Median	25.5
IQR	9–41
Surviving patients (n = 12)	
Median	45.5
IQR	33–54
**Time between surgery and therapy initiation (months)**	
Median	7
IQR	2–36
**Duration of sunitinib treatment (months)**	
Median	11.1
IQR	6–24

1 two patients were excluded due to missing follow up data.

### DNA Extraction from Blood and Tumor Tissues

The isolation of lymphocytes from 22 healthy donors from approximately 9 ml peripheral blood was performed with 15 ml Biocoll Separating Solution (BIOCHROM AG; Berlin, Germany) and a density gradient. Afterwards the lymphocytes were washed with PBS two times. Genomic DNA was isolated from lymphocytes using the Invisorb Spin Cell Mini Kit (STRATEC Molecular GmbH; Berlin, Germany) according to the manufacturer’s protocol.

Cryo-preserved malignant and corresponding non-malignant tissue specimens from 20 primary ccRCC were used for DNA extraction. DNA isolation from cryosectioned tissue was accomplished with the Invisorb Spin Tissue Mini Kit (STRATEC Molecular GmbH) according to the manufacturer’s recommendations. The concentrations and purity of DNA was measured on a NanoDrop 1000 (PEQLAB; Erlangen, Germany).

### Copy Number Analysis for VHL Gene

A total of 25 ng of genomic DNA from 20 tumor tissues and of lymphocytic DNA from 22 healthy donors were used for copy number analysis by quantitative PCR (qPCR). The PCR amplification reaction was performed with the TaqMan Copy Number Assays for VHL (Hs06700943-cn) and RNase P (Life Technologies GmbH, Darmstadt, Germany) following the manufacturer’s instructions. The target gene VHL and the reference gene RNase P were amplified on a LC480 Real-Time PCR system (Roche; Mannheim, Germany) and quantified by the ΔΔCT method for calculation of gene copy number in the tumor tissue relative to lymphocyte DNA of healthy donors.

### VHL Mutation Analysis

Tumor DNA was amplified with five pairs of VHL-specific primers (Table 3) for mutational analysis. The two primers used for amplification of exon 1 and flanking regions were described previously [33]. Primers for exons 2, 3a and 3b were designed by the Primer Select software of DNASTAR Lasergene 8 (DNASTAR, Inc, Madison, USA). PCR was performed in 50 µl reaction mixtures consisting of 100 ng template DNA, 1 µM of each primer and 2.5 U HotStar HiFidelity polymerase (Qiagen GmbH; Hilden, Germany). Reaction mixtures were incubated at 95°C for 5 min followed by 35 cycles of a three-step PCR (15 s at 94°C, 60 s at 60°C and 60 s at 72°C) and 10 min at 72°C. The amplification products were checked for size and purity by agarose gel electrophoresis. The QIAquick PCR Purification Kit (Qiagen) was used to retrieve the remaining PCR product. The retrieved product was subsequently treated with exonuclease (New England Biolabs GmbH; Frankfurt am Main, Germany) and alkaline phosphatase (Roche) to remove leftover primers and dNTPs. VHL mutation analysis was done by direct sequencing using the BigDye Terminator v1.1 Cycle Sequencing Kit (Life Technologies GmbH) according to the manufacturer’s protocol. Reactions were carried out for 25 cycles using a MJ-Research PTC-100 Thermal Controller (Global Medical Instrumentation Inc.; Ramsey, USA) and sequencing was performed by fluorescence capillary electrophoresis using an ABI 3100 Genetic Analyzer (Life Technologies GmbH). Data were processed using Sequencing Analysis software (Life Technologies GmbH) and by visual inspection of electropherograms.

**Table 3:** Primer for VHL mutation analyses.

VHL gene region	Primer sequence	Annealing temperature (°C)	Product size (bp)
**Exon 1a**	CGCGAAGACTACGGAGGT (sense)	60	348
	GGACTGCGATTGCAGAAGAT (antisense)		
**Exon 1b**	GAGTACGGCCCTGAAGAAGA (sense)	60	332
	GCTTCAGACCGTGCTATCGT (antisense)		
**Exon 2**	ACCGGTGTGGCTCTTTAACAACCT (sense)	60	376
	GCCCAAAGTGCTTTTGAGACACCA (antisense)		
**Exon 3a**	GCCTCTTGTTCGTTCCTTGTACTGA (sense)	60	574
	ACGATATGCTGCAATTCCCACTGAA (antisense)		
**Exon 3b**	GAAATTACTACAGAGGCATGAACACCAT (sense)	60	510
	GTGCCTATTTTACTCTGAGAATGAGACACT (antisense)		

### Tissue Microarray (TMA) Generation

Formalin-fixed paraffin-embedded (FFPE) ccRCC and corresponding non-malignant tissue specimens from 42 patients were histopathologically examined for tumor stage, Fuhrman grade and TNM staging system according to International Union Against Cancer (UICC 2003 and 2010) by an experienced pathologist. After review of the hematoxylin and eosin staining of the specimen sections tissue cylinders (600 µm in diameter or 0.283 mm^2^) were taken from the tumor and non-tumor regions in the paraffin donor blocks and assembled in an array-like format into an empty acceptor block [34]. Five tissue microarrays (TMA) with 144–160 tissue cores per array were constructed and each case was represented by three tissue cores of malignant and corresponding non-malignant tissue.

### Immunohistochemistry

Immunohistochemistry (IHC) was performed on 600 µm TMA sections with the antibodies and antigen retrieval methods listed in Table S1. The peroxidase-labled streptavidin-biotin system (Vector Laboratories; Burlingame, USA) with 3.3-diaminobenzidine (Dako Deutschland GmbH; Hamburg, Germany) as chromagen was used to visualize the bound antibodies except for those against CD31, CD34 and Ki67. For these three antibodies the UltraVision LP detection system with HRP Polymer (Thermo Scientific; Fremont, USA) was used (Table S1). Positive and negative controls were run simultaneously and showed appropriate immunostaining. The immunohistochemical staining was assessed in different categories such as percentage of stained cells (0–100%), staining intensity (0–3), cell membrane staining (yes = 1 or no = 0), number of stained nuclei, vessel (endothelial) staining and MVD. MVD was calculated as the ratio of the total number of either CD31 or CD34 stained vessels and tissue core area (0.283 mm^2^). The score is the product of the percentage of stained cells and staining intensity.

### Statistical Design and Data Analysis

Follow-up data, PFS and OS were registered from medical records and by contacting patients’ urologists and oncologists. PFS as the primary endpoint was defined as time between the first day of sunitinib treatment and date of progressive disease (PD) according to the RECIST criteria or date of death after the last response evaluation. Most patients with PD switched to another therapeutic such as sorafenib, everolimus or temsirolimus. In this case patients were excluded from further PFS analyses at the subsequent time points of treatment evaluation. If a patient did not progress, PFS was censored at the time of last follow-up. OS as the secondary endpoint was calculated from date of sunitinib initiation until date of death or last known date of patients being alive.

Statistical analyses (Mann Whitney-U test, Spearman correlation, Fisher’s exact test, Kaplan-Meier method, log-rank test and Cox regression) were done by SPSS statistics (version 19). A p-value <0.05 was defined to be statistically significant; p<0.1 was considered as a statistical trend. The clinicopathological parameters were categorized for all analyses as follows: pT stage into organ confined disease (OCD; pT1 and 2) and non-organ confined disease (NOCD; pT3 and 4), Fuhrman grading into “low” (grade 1 and 2) and “high” (grade 3 and 4), primary lymph node status and distant metastasis into “negative” (M0/N0) and “positive” (M1/N+) and TNM stage into ”low” (stage I and II) and “high” (stage III and IV). For survival analyses patients were dichotomized at the median immunoexpression of the markers into groups with low and high expression except for the vessel staining of VEGFRs and PDGFRs as well as the membrane staining of CA9. These variables were categorized into “low” (<1) and “high” ( = 1).

## Results

### Patients and Follow-up

Approximately 30% of the 42 ccRCC patients (with a median age of 64 years at surgery) had an OCD, whereas a NOCD was diagnosed in nearly 70% at time of surgery (Table 1). Twenty eight patients (66.7%) had primary lymph node or distant metastasis at the time of initial diagnosis and surgery. Within a median follow-up of 39.6 months (interquartile range (IQR) 13–45 months) 28 (66.7%) patients died of disease (Table 2). The median duration of sunitinib treatment was 11.1 months (IQR 6-24 months) and therapy started in median 7 months (IQR 2-36 months) after nephrectomy.

### Association of Potential Markers with Clinicopathological Parameters

The raw data for each patient and representative staining images of each antibody are summarized in Table S2 and Figure S1. The assessment of immunohistochemical staining showed significant associations between clinicopathological parameters and different molecular markers such as CD31, CD34, HIF-1α, VEGFR1, -2 and -3, pVEGFR1 and -2, PDGFRα and -β, pPDGFRα and -β, Ki67 and SVV using the evaluation criteria staining score and intensity, stained nuclei, vessel staining and MVD (Table 4). Particularly numerous associations of markers with Fuhrman grading as well as with primary lymph node and distant metastasis were observed. These two parameters demonstrated frequently significant associations with VEGFR1, -2 and -3 as well as pPDGFRα and -β. Higher mean expression of CD31 and CD34 reflecting MVD, HIF-1α score, VEGFR1, -2, and -3 vessel staining, pVEGFR1 and -2 score and intensity as well as pPDGFRα and -β score and intensity was observed in the categories of the clinicopathological parameters with improved outcome (OCD, low grade, M0/N0 and low TNM). In contrast, the nuclear markers Ki67 and SVV showed higher protein levels in the categories NOCD, high grade, M1/N+ and high TNM (Table 4). Furthermore, VEGFR1 and -2 score and intensity as well as PDGFRα and -β score, intensity and vessel staining revealed higher mean expression in tumor tissues from patients with clinical parameters of poor prognosis. No significant results were observed for VEGFA, its co-receptor NRP-1 and the anti-angiogenic isoform VEGFA_165_B.

**Table 4:** Significant associations of protein expression of potential markers with clinicopathological parameters.

Parameter	Potential marker	Subgroup	Mean expression^1^	Subgroup	Mean expression^1^	p-value^2^
**pT**	PDGFRα intensity	**OCD**	0.83 (14/42)	**NOCD**	1.12 (28/42)	0.027
	VEGFR1 vessel staining		0.88 (14/41)		0.66 (27/41)	0.046
**Fuhrmann grading**	CD31 MVD	**low (1+2)**	155.2 (21/42)	**high (3+4)**	86.3 (21/42)	0.018
	CD34 MVD		178.8 (21/41)		99.3 (20/41)	0.027
	HIF-1α score		74.40 (21/41)		50.66 (20/41)	0.021
	VEGFR1 score		104.2 (21/41)		141.6 (20/41)	0.020
	VEGFR1 intensity		1.18 (21/41)		1.51 (20/41)	0.016
	VEGFR1 vessel staining		0.83 (21/41)		0.63 (20/41)	0.020
	VEGFR2 vessel staining		0.88 (21/42)		0.62 (21/42)	0.015
	VEGFR3 vessel staining		0.62 (21/42)		0.31 (21/42)	0.022
	pPDGFRα score		85.7 (21/42)		26.9 (21/42)	0.000
	pPDGFRα intensity		1.09 (21/42)		0.51 (21/42)	0.001
	pPDGFRβ score		86.3 (21/42)		43.7 (21/42)	0.002
	pPDGFRβ intensity		0.99 (21/42)		0.65 (21/42)	0.015
**M/N combined**	PDGFRβ vessel staining	**M0/N0**	0.17 (14/41)	**M1/N+** 3	0.55 (27/41)	0.006
	VEGFR1 intensity		1.16 (14/41)		1.44 (27/41)	0.029
	VEGFR2 score		48.0 (14/41)		86.8 (28/41)	0.042
	VEGFR2 intensity		0.79 (14/42)		1.19 (28/42)	0.036
	pVEGFR1 score		44.0 (14/42)		18.5 (28/42)	0.043
	pVEGFR1 intensity		0.66 (14/42)		0.32 (28/42)	0.046
	pVEGFR2 score		45.0 (14/42)		17.5 (28/42)	0.044
	pPDGFRα score		77.6 (14/42)		48.1 (28/42)	0.044
	pPDGFRα intensity		1.05 (14/42)		0.70 (28/42)	0.045
	pPDGFRβ score		93.7 (14/42)		53.0 (28/42)	0.018
	pPDGFRβ intensity		1.11 (14/42)		0.71 (28/42)	0.010
	Ki67 stained nuclei		1.40 (14/42)		6.66 (28/42)	0.005
	SVV stained nuclei		5.8 (14/40)		14.0 (26/40)	0.005
**TNM staging**	PDGFRα score	**low (I+II)**	35.9 (7/42)	**high (III+IV)**	70.44 (35/42)	0.045
	PDGFRα intensity		0.76 (7/42)		1.07 (35/42)	0.025
	Ki67 stained nuclei		1.10 (7/42)		5.38 (35/42)	0.031

OCD: organ confined disease; NOCD: non-organ confined disease.

1 the numbers in brackets represent the numbers of patients in each group in relation to all evaluable patients.

2 Mann-Whitney U-test.

3 Patients had distant metastasis and/or lymph node metastasis at time of nephrectomy.

### Response to Sunitinib Treatment and Association with Adverse Effects and Molecular Markers

The objective response rate to sunitinib therapy was evaluated after 3, 6 and 9 months after therapy start as well as the after last report (range 12–54 months) from 38 patients with available response information (Table 5). For example: a response after 9 months was assessed in 21 (55.3%) of all treated patients. At this time adverse effects under treatment like HTN and HFS were observed in 13 and 11 patients, respectively, out of 24 patients with available data. Significant associations between sunitinib response and HTN as well as HFS were not found. Nevertheless, responders showed a trend to exhibit HTN (p = 0.067) after last report (Table 5). The response after 9 months was significantly associated with low Fuhrman grade (p = 0.010; data not shown).

**Table 5:** Response and adverse effects after different periods of sunitinib treatment.

Parameter	Response after 3 months^1^	Response after 6 months^1^	Response after 9 months^1^	Response after last report^1^
**Response (n** = **38)**				
** responders**	32 (84.2%)	27 (71.1%)	21 (55.3%)	10 (26.3%)
partial response	10 (26.3%)	6 (15.8%)	4 (10.5%)	1(2.6%)
mixed response	2 (5.3%)	1 (2.6%)	-	-
stable disease	20 (52.6%)	20 (52.6%)	17 (44.7%)	9 (23.7%)
**non-responders**	2 (5.3%)	6 (15.8%)	4 (10.5%)	7 (13.9%)
**Adverse effects**				
** Hand-foot syndrome**				
** Responders**	(n = 18)	(n = 15)	(n = 13)	(n = 5)
Yes	11	9	8	2
No	7	6	5	3
** Non-Responders**	(n = 0)	(n = 3)	(n = 2)	(n = 3)
Yes	0	1	1	2
No	0	2	1	1
** Hypertension**				
** Responders**	(n = 20)	(n = 17)	(n = 15)	(n = 7)
Yes	11	10	9	7
No	9	7	6	0
** Non-Responders**	(n = 1)	(n = 4)	(n = 2)	(n = 3)
Yes	1	3	0	1
No	0	1	2	2

1 percentage of responders and non-responders in relation to all patients with available response data (n = 38).

Potential predictive markers such as CA9, CD31, HIF-1α, pVEGFR1, pPDGFRα and -β, Ki67 and VEGFR1 and -2 displayed significant associations with a response to sunitinib treatment (Table 6). Interestingly, the response after 6 months was related to markers responsible for pH regulation (CA9) and vessel formation (CD31) whereas after last report VEGF receptors might be more important. In addition, trends (p<0.1) were evidenced for CD34 (response after 3 months), CA9, VEGFR1 and SVV (response after 6 months), pPDGFRβ and SVV (response after 9 months) and VEGFR1 (after last report) (data not shown).

**Table 6:** Association of expression of potential markers with a sunitinib response.

Response	Potential marker (n = responders + non-responders)	Mean expression responders	Mean expression non-responders	p-value^1^
**after 6 months**	CA9 intensity (n = 27+6)	2.37	1.53	0.034
	CD31 intensity (n = 27+6)	2.14	1.69	0.049
**after 9 months**	HIF-1α score (n = 20+4)	75.1	13.3	0.021
	pVEGFR1 stained nuclei (n = 21+4)	48.2	4.79	0.038
	pPDGFRα score (n = 21+4)	81.5	6.07	0.012
	pPDGFRα intensity (n = 21+4)	1.03	0.16	0.011
	pPDGFRβ score (n = 21+4)	81.5	26.1	0.041
	Ki67 stained nuclei (n = 21+4)	2.04	16.1	0.004
**last report** 2	VEGFR1 intensity (n = 10+6)	1.01	1.41	0.003
	VEGFR1 vessel staining (n = 10+6)	0.90	0.76	0.048
	VEGFR2 vessel staining (n = 10+7)	0.95	0.53	0.010

1 Mann-Whitney U-test.

2 last report means 12 - 54 months.

### Survival Data in Relation to Potential Molecular Markers and Clinicopathological Parameters

**Figure 1:**
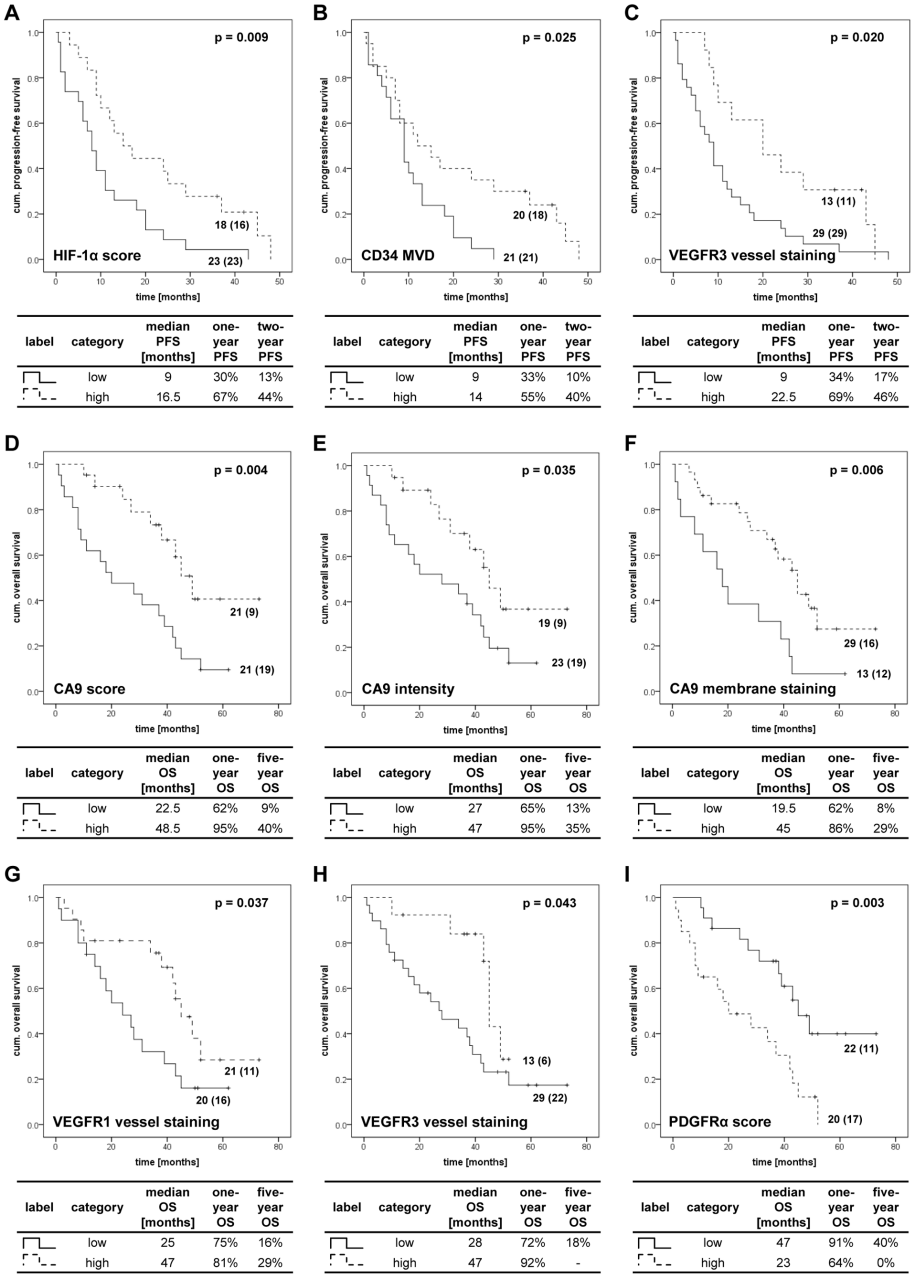
Kaplan-Meier plots for IHC-markers with significant associations to PFS and OS. Significant differences in PFS (log-rank test) were observed for HIF-1α score (A), CD34 MVD (B) and VEGFR3 vessel staining (C). CA9 score (D), CA9 intensity (E) and CA9 membrane staining (F), VEGFR1 vessel staining (G), VEGFR3 vessel staining (H) and PDGFRα score (I) were significantly associated with OS. The data at each curve represent the number of patients per subgroup; the number of events for each subgroup is shown in brackets. The table under each Kaplan-Meier plot contains the median PFS or OS as well as the one-, two- or five-year PFS or OS for this marker.

By using the Kaplan-Meier method all molecular markers and clinicopathological parameters were investigated with regard to their association with PFS and OS. Significant associations with longer PFS were identified for high HIF-1α score, CD34 MVD and VEGFR3 vessel staining (Figure 1). Other markers such as a high CA9 score, intensity and membrane staining, VEGFR1 and -3 vessel staining and low PDGFRα score were significantly associated with longer OS (Table 7). The median OS was for example 22.5 months for patients (n = 19) with a low and 48.5 months for patients (n = 9) with a high CA9 score. After 5 years, 9% of patients with low and 40% of the patients with a high CA9 score were still alive. There was an association by trend between the combined M/N status and PFS (p = 0.061) as well as between Fuhrman grading and OS (p = 0.091) as assessed by log-rank test. Other clinico-pathological parameters were not related to differences in OS and PFS (data not shown). The same molecular markers also displayed significant results in univariate analyses for PFS and OS (Table 7). Clinicopathological parameters (Fuhrman grading and M/N combined) demonstrated only trends in univariate analyses. Multivariate analyses showed that CA9 membrane staining was an independent prognostic marker for OS (HR 0.174, 95%-CI 0.045-0.669, p = 0.011).

**Table 7:** Univariate and multivariate analysis of clinicopathologic parameters and potential markers with regard to PFS and OS^1^.

Parameter (n)	Distribution (low^2^ + high)	Statistical parameter	Univariate analysis PFS	Univariate analysis OS	Multivariate analysis^3^ OS
**Fuhrman grading (42)**	21 + 21	HR		1.913	
		95%-CI		0.902–4.059	
		p-value		0.091	
**M/N combined (42)**	14 + 28	HR	1.946		
		95%-CI	0.970–3.903		
		p-value	0.061		
**CA9 score (42)**	21 + 21	HR		**0.335**	
		95%-CI		**0.151**–**0.742**	
		p-value		**0.007**	
**CA9 intensity (42)**	23 + 19	HR	0.559	**0.437**	
		95%-CI	0.294-1.062	**0.197**–**0.969**	
		p-value	0.076	**0.042**	
**CA9 membrane staining (42)**	13 + 29	HR		**0.359**	**0.174**
		95%-CI		**0.168**–**0.767**	**0.045**–**0.669**
		p-value		**0.008**	**0.011**
**CD34 MVD (41)**	21 + 20	HR	**0.466**		
		95%-CI	**0.232**–**0.938**		
		p-value	**0.033**		
**HIF-1α score (41)**	23 + 18	HR	**0.422**		
		95%-CI	**0.214**–**0.832**		
		p-value	**0.011**		
**PDGFRα score (42)**	22 + 20	HR	1.758	**3.000**	
		95%-CI	0.927–3.334	**1.393**–**6.459**	
		p-value	0.084	**0.005**	
**VEGFR1 score (41)**	21 + 20	HR	1.731		
		95%-CI	0.906–3.306		
		p-value	0.097		
**VEGFR1 intensity (41)**	21 + 20	HR	1.843		
		95%-CI	0.963–3.524		
		p-value	0.065		
**VEGFR1 vessel staining (41)**	20 + 21	HR		**0.449**	
		95%-CI		**0.206**–**0.976**	
		p-value		**0.043**	
**VEGFR3 vessel staining (42)**	29 + 13	HR	**0.447**	0.407	
		95%-CI	**0.220**–**0.909**	0.164–1.009	
		p-value	**0.026**	0.052	
**SVV stained nuclei (40)**	20 + 20	HR	1.782		
		95%-CI	0.921–3.450		
		p-value	0.086		

1 table contains trends and significant results (in bold) of univariate and multivariate analysis of OS and PFS.

2 low is reference category and set as HR = 1.

3 only significant parameters from the univariate analyses of table 7 and 8 were included in the multivariate analyses.

### Survival Data in Relation to Sunitinib Response Prediction

**Figure 2:**
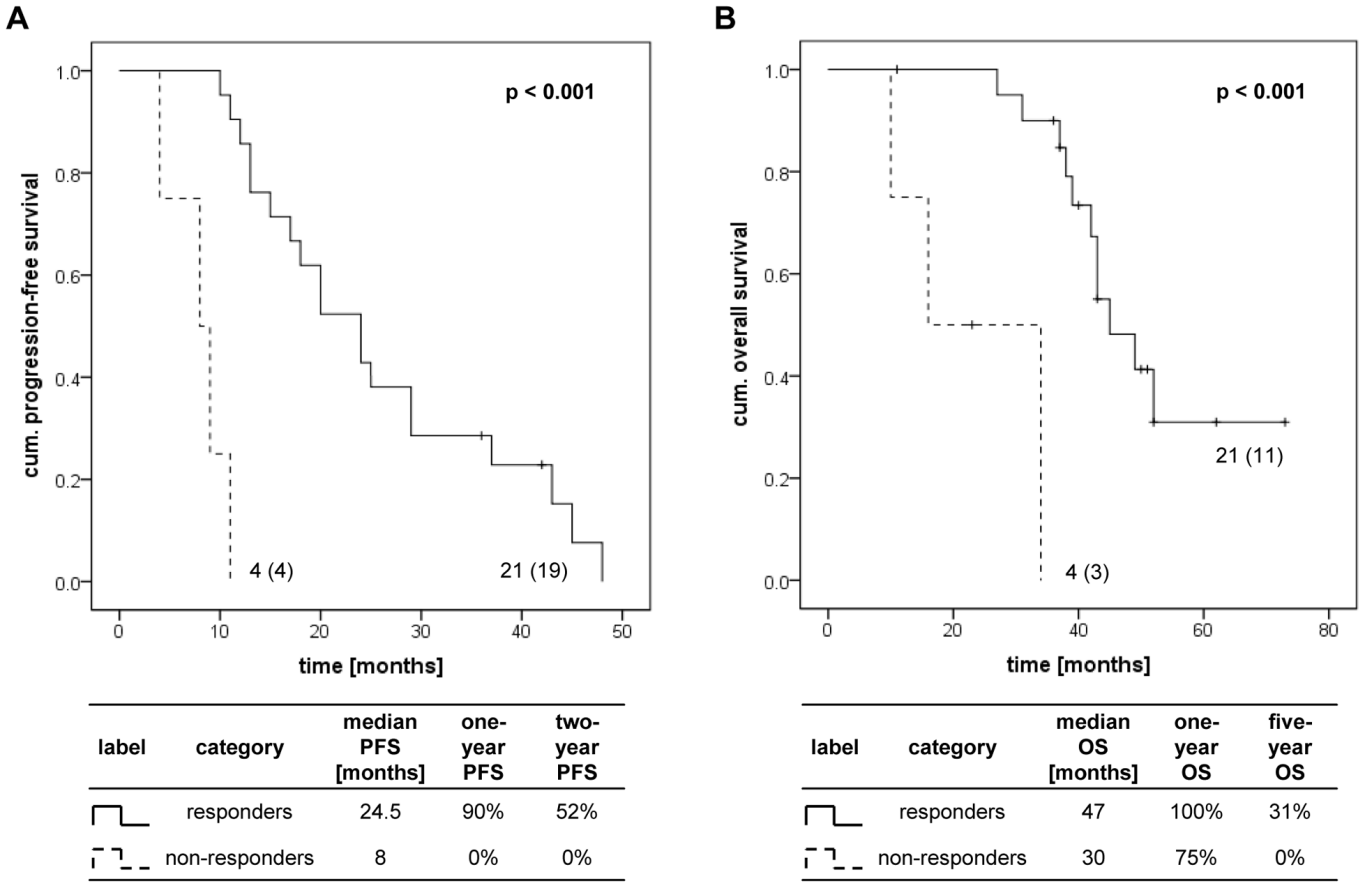
Kaplan-Meier plots for response to sunitinib treatment after 9 months. Differences in PFS (A) and OS (B) with regard to patients’ response to sunitinib therapy were calculated by the log-rank test. The data at each curve represent the number of patients per subgroup; the number of events for each subgroup is shown in brackets. The table under each Kaplan-Meier plot contains the median PFS or OS as well as the one-, two- or five-year PFS or OS in dependence on the response to sunitinib treatment after 9 months.

Different response rates after 9 months indicated significant differences in PFS and OS (log-rank test p<0.001) as shown in Kaplan-Meier analyses (Figure 2). Responders had an one-year PFS of 90% whereas it was 0% for non-responders. Also the OS showed differences for responders and non-responders after 9 months with survival rates of 100% and 75% after one year, respectively. Comparison between responders and non-responders revealed significant differences in median PFS (24.5 versus 8 months) and in median OS (47 versus 30 months) (Figure 2). A response after 9 months had a significant influence on PFS (HR 0.039, 95%-CI 0.007–0.224, p<0.001) and OS (HR 0.054, 95%-CI 0.008–0.344, p = 0.002) as shown in univariate Cox regression analyses (Table 8). Similar results were obtained for a response after 6 months albeit reaching significance only for PFS. In multivariate Cox regression analyses a response after 9 months emerged as an independent prognostic marker for OS (HR 0.038, 95%-CI 0.005–0.299, p = 0.002) (Table 8).

**Table 8:** Univariate and multivariate analysis of response to sunitinib-treatment with regard to PFS and OS^1^.

Response after (n)	Distribution (responder + non-responder^2^)	Statistical parameter	Univariate analysis PFS	Univariate analysis OS	Multivariate analysis^3^ OS
**6 months (33)**	27 + 6	HR	**0.149**	0.366	
		95%-CI	**0.047**–**0.473**	0.130–1.028	
		p-value	**0.001**	0.056	
**9 months (25)**	21 + 4	HR	**0.039**	**0.054**	**0.038**
		95%-CI	**0.007**–**0.224**	**0.008**–**0.344**	**0.005**–**0.299**
		p-value	**<0.001**	**0.002**	**0.002**

1 table contains trends and significant results (in bold) of univariate and multivariate analysis of OS and PFS.

2 non-responder is reference category and set as HR = 1.

3 only significant parameters from the univariate analyses of table 7 and 8 were included in the multivariate analyses.

### Multivariate Cox Proportional Hazards Regression Models for PFS and OS

Multivariate Cox proportional hazards regression models were generated for analysis of PFS and OS of ccRCC patients treated with sunitinib. Clinicopathological parameters such as pT, G and M/N that characterize the primary tumor were included to the basic model. In the next step IHC-markers and response variables were separately added to the model to validate them as potential independent prognostic parameters. HIF-1α score (HR 0.456, 95%-CI 0.227–0.916, p = 0.027), VEGFR3 vessel staining (HR 0.396, 95%-CI 0.163–0.964, p = 0.041), a response after 6 months (HR 0.175, 95%-CI 0.054–0.570, p = 0.004), after 9 months (HR 0.020, 95%-CI 0.002–0.162, p<0.001) and after the last report (HR 0.170, 95%-CI 0.034–0.862, p = 0.032) were significant parameters for PFS (Table 9). Potential markers such as CA9 score (HR 0.271, 95%-CI 0.110–0.669, p = 0.005), CA9 intensity (HR 0.428, 95%-CI 0.184–0.992, p = 0.048) and CA9 membrane staining (HR 0.356, 95%-CI 0.157–0.807, p = 0.013), VEGFR3 vessel staining (HR 0.323, 95%-CI 0.112–0.934, p = 0.037), PDGFRα score (HR 3.375, 95%-CI 1.414–8.056, p = 0.006), a response after 6 months (HR 0.265, 95%-CI 0.080–0.877, p = 0.030) and after 9 months (HR 0.033, 95%-CI 0.003–0.373, p = 0.006) exhibited significant associations with OS (Table 10).

**Table 9:** Multivariate Cox proportional hazard model for PFS^1^.

Factor	Statistical parameter	Model 1	Model 1+ HIF-1α score	Model 1+ VEGFR3 vessel staining	Model 1+ response after 6 months	Model 1+ response after 9 months	Model 1+ last report
**pT**	HR	1.363	1.553	0.943	1.685	2.525	2.398
	95%-CI	0.614–3.029	0.664–3.636	0.386–2.307	0.664–4.274	0.857–7.440	0.643–8.942
	p-value	0.447	0.310	0.898	0.272	0.093	0.193
**G**	HR	1.329	1.036	1.1016	1.040	**0.248**	**0.072**
	95%-CI	0.653–2.705	0.486–2.206	0.470–2.193	0.461–2.349	**0.064**–**0.960**	**0.008**–**0.626**
	p-value	0.432	0.927	0.969	0.925	**0.044**	**0.017**
**N/M**	HR	1.814	1.680	**2.207**	1.795	1.568	1.208
	95%-CI	0.890–3.700	0.809–3.486	**1.038**–**4.692**	0.829–3.888	0.606–4.058	0.388–3.754
	p-value	0.101	0.164	**0.040**	0.138	0.354	0.745
**HIF-1α score**	HR		**0.456**				
	95%-CI		**0.227**–**0.916**				
	p-value		**0.027**				
**VEGFR3 vessel staining**	HR			**0.396**			
	95%-CI			**0.163**–**0.964**			
	p-value			**0.041**			
**response after 6 months**	HR				**0.175**		
	95%-CI				**0.054**–**0.570**		
	p-value				**0.004**		
**response after 9 months**	HR					**0.020**	
	95%-CI					**0.002**–**0.162**	
	p-value					**<0.001**	
**last report**	HR						**0.170**
	95%-CI						**0.034**–**0.862**
	p-value						**0.032**

1 table contains all significant (in bold) molecular markers and responses.

**Table 10:** Multivariate Cox proportional hazard model for OS^1^.

Factor	Statistical parameter	Model 1	Model 1 + CA9 score	Model 1 + CA9 intensity	Model 1 + CA9 membrane staining	Model 1 + VEGFR3 vessel staining	Model 1 + PDGFRα score	Model 1 + response after 6 months	Model 1 + response after 9 months
**pT**	HR	0.685	0.482	0.639	0.514	0.464	0.473	0.601	0.993
	95%-CI	0.283–1.661	0.185–1.253	0.267–1.534	0.196–1.346	0.181–1.191	0.193–1.158	0.211–1.716	0.302–3.265
	p-value	0.403	0.134	0.316	0.176	0.111	0.101	0.342	0.991
**G**	HR	1.977	**2.504**	2.136	1.788	1.449	1.548	2.457	0.537
	95%-CI	0.871–4.489	**1.066**–**5.882**	0.932–4.896	0.760–4.209	0.598–3.510	0.700–3.560	0.960–6.293	0.113–2.566
	p-value	0.103	**0.035**	0.073	0.183	0.411	0.271	0.061	0.436
**N/M**	HR	1.532	0.940	1.175	1.466	1.876	1.448	2.065	1.017
	95%-CI	0.649–3.619	0.364–2.428	0.471–2.931	0.600–3.584	0.758–4.645	0.616–3.401	0.811–5.258	0.305–3.396
	p-value	0.331	0.899	0.730	0.401	0.174	0.396	0.128	0.978
**CA9 score**	HR		**0.271**						
	95%-CI		**0.110**–**0.669**						
	p-value		**0.005**						
**CA9 intensity**	HR			**0.428**					
	95%-CI			**0.184**–**0.992**					
	p-value			**0.048**					
**CA9 membrane staining**	HR				**0.356**				
	95%-CI				**0.157**–**0.807**				
	p-value				**0.013**				
**VEGFR3 vessel staining**	HR					**0.323**			
	95%-CI					**0.112**–**0.934**			
	p-value					**0.037**			
**PDGFRα score**	HR						**3.375**		
	95%-CI						**1.414**–**8.056**		
	p-value						**0.006**		
**response after 6 months**	HR							**0.265**	
	95%-CI							**0.080**–**0.877**	
	p-value							**0.030**	
**response after 9 months**	HR								**0.033**
	95%-CI								**0.003**–**0.373**
	p-value								**0.006**

1 table contains all significant (in bold) molecular markers and responses.

### VHL Copy Number and Mutation Analysis

**Figure 3:**
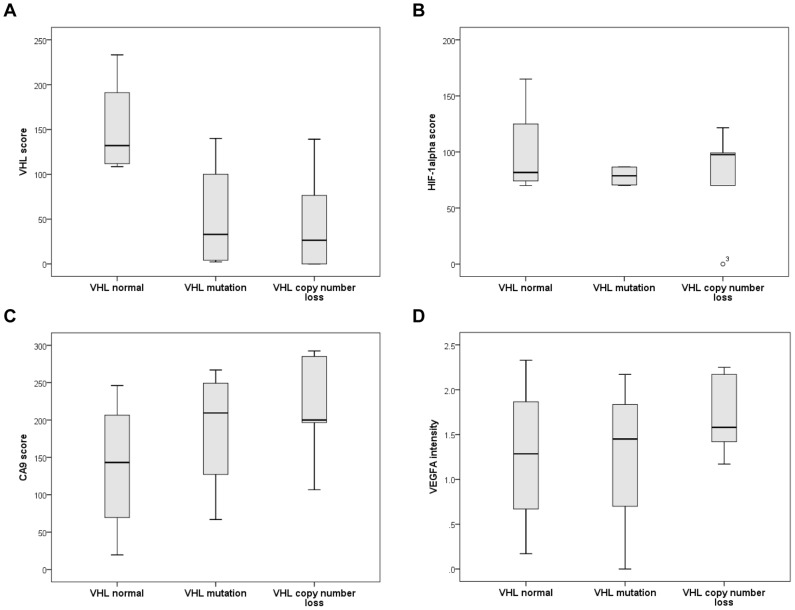
Effect of VHL gene alterations on protein expression of VHL and its target genes. Boxplots show the VHL score (A), HIF-1α score (B), CA9 score (C) and VEGFA intensity (D) of patients with normal VHL status, with VHL mutations and copy number losses.

These functional analyses revealed a VHL copy number loss in 60% of 20 evaluable, sunitinib-treated ccRCC patients and mutations of the VHL gene were detected in 50% of these patients (raw data for each patient see in Table S2). One non-sense, 2 missense and 7 frameshift mutations were determined in the three exons of the VHL gene. Both VHL alterations (copy number and mutation) were observed simultaneously in 30% of the cases. Furthermore, the results of VHL changes were compared with immunohistochemical stainings of VHL and its target genes HIF-1α, CA9 and VEGFA confirming the suggested causal chain. The VHL score declined and CA9 score was increased in patients with mutation and copy number loss (Figure 3). However, only a slight rise could be observed for HIF-1α score and VEGFA intensity in patients with VHL alterations in contrast to those with normal VHL status (Figure 3).

## Discussion

Metastatic ccRCC patients urgently need molecular markers and models for the prediction of a response to TKI treatment. Presently there are very few and limited studies each analyzing only one marker. Therefore, we aimed at a different, more comprehensive approach. For this purpose, we systematically evaluated factors involved in angiogenic pathways in primary tumor tissues from patients with metastatic ccRCC who were treated with sunitinib. Starting with VHL and going on with its target genes HIF-1α, VEGFA, VEGFA_165_B (as an anti-angiogenic isoform of VEGFA) and CA9, we also assessed immunoexpression of different endothelial markers such as CD31, CD34, VEGFR1, -2 and -3, pVEGFR2, NRP-1, PDGFRα and -β and pPDGFRα and -β as well as of the well-known prognostic markers Ki67 and SVV. This is the first study investigating IHC staining of the active, phosphorylated VEGF and PDGF receptors (pVEGFR1 and -2, pPDGFRα and -β) in relation to sunitinib response. As mentioned before, our study was based on analyzing primary tumor tissue, although only the development of metastases required a TKI therapy. However, metastatic tissue is rarely available for such analyses. Since nearly 30% of patients showed metastases at tumor diagnosis and another 30% develop them later, we used primary tumors, which are usually available after tumor nephrectomy for immunohistochemical staining of potential predictive molecular markers [35]. IHC is a well-established method that could be easily transferred into practice for prognostic and predictive purposes. The immunoexpression of potential molecular markers and the aggressiveness of the primary tumor are assumed to support the prediction of a response to sunitinib after patients develop metastasis. Ongoing large biomarker studies are not yet completed, but the results are expected to enable the response prediction to TKI treatment.

The patient cohort of the present study included 42 cases, 69% males and 31% females, with a median age at initiation of therapy of 67 years. Other studies identified similar data of patients’ age, gender and their distribution in clinicopathological parameters such as pT stage and Fuhrman grade [21], [36]. Therefore, the patient cohort analyzed in this study might be representative despite of the rather small number of cases. The median PFS and OS of all patients was 10.5 and 35 months, respectively, and comparable to the median PFS of 11 months and median OS of 26.4 months in the subset of 375 sunitinib-treated patients in the study by Motzer *et al.* [4]. In accordance with our results the study by Choueiri *et al.* also demonstrated a median PFS and OS of 10.8 and 29.8 months, though their patients received different VEGF targeted therapies (63% sunitinib, 28% sorafenib, 14% axitinib and 17% bevacizumab) [37].

The assessment of immunohistochemical staining defined several correlations between the expression of molecular makers (e.g. HIF-1α, CD31, CD34, VEGFR1, -2 and -3, pVEGFR1, (p)PDGFRα and -β, Ki67 and SVV) and the distribution of clinicopathological parameters like pT stage, Fuhrman grading, combined M/N and TNM stage. Other studies also revealed significant associations between tumor expression of HIF-1α as well as SVV in tumor tissue and pT [38], [39]. In the present study there were significant associations between HIF-1α and Fuhrman grading as well as between SVV and combined M/N stage. In contrast to our data the study by Yilmazer *et al.* showed significant correlations between CD34 as well as VEGF and pT and TNM stage, respectively [40]. However, no information on Fuhrman grade evaluation was provided in these studies. Moreover, Bui *et al.* demonstrated a significant association of Ki67 with pT, Fuhrman grade, nodal and metastatic status [41], whereas our results identified a significant association of Ki67 with TNM stage, which includes pT as well as nodal and metastatic status. These data support the hypothesis that some molecular markers could be potentially used as a prognostic marker. According to the EAU Guidelines (2012) TNM and Fuhrman grading were only included in prognostic models for local RCC, but not for metastatic RCC.

Furthermore, this study focused on identifying new and evaluating known molecular markers for prediction of a sunitinib response. In the present study, patients displayed objective response rates of nearly 55% after 9 months and around 26% after the last report. A previous study by Choueiri *et al.* determined an objective response rate of 37%, including only partial response, which shows similarity with our partial response rate of 26% after 3 months [42]. Another study by Motzer *et al.* reported that 40% and 27% of sunitinib treated patients displayed a partial response and a stable disease, respectively [5], which seems to be comparable to the response after 6 months (71%) of this study. Possible discrepancies between our evaluated response rates and results of these studies can mainly be attributed to the differences in size of the patient cohorts and the different periods of response evaluation.

The intake of TKIs like sunitinib is known to lead to the occurrence of adverse effects (grade 3) such as fatigue, thrombocytopenia, anaemia, HFS, HTN and leukopenia, which are reversible after therapy discontinuation [43]. Most studies demonstrated significant associations between HTN as well as HFS and better response rates to sunitinib, longer PFS and OS [44], [45]. In this study, an assessment of adverse effects of sunitinib treatment revealed that 54% and 46% of the patients developed HTN and HFS, respectively. However, the present study identified no statistical significant associations between these adverse events and PFS or OS but we observed an association by trend between HTN and response after the last report (p = 0.067).

Data collection of adverse events is important but insufficient for response prediction and therefore, identification of further markers that enable response prediction is essential for metastatic ccRCC patients. Validation of already shown associations between molecular markers and sunitinib response was also an important part of this study. In agreement with our results Terakawa *et al.* demonstrated a significant relation between response to sunitinib and tumor grade, even though they used the three-stage grading, as well as with strong VEGFR2 expression [21]. In addition, selective inhibition of VEGFR2 kinase activity by sunitinib was higher than those of other kinases [46]. Therefore, both findings support the hypothesis that patients with higher VEGFR2 expression in the primary tumor show greater clinical benefits from sunitinib treatment. Another study described high levels of HIF-1α being significantly associated with an improved objective clinical response of metastatic ccRCC patients to sunitinib [20]. High expression of HIF-1α was also associated with a good response after 9 months in the present study. Moreover, we observed that the HIF-1α regulated transmembrane protein CA9 was significantly increased in patients responding to sunitinib therapy after 6 months. Similar data revealed associations of tumor responsiveness between high CA9 expression and anti-VEGF therapy agents such as sunitinib, sorafenib, bevacizumab, temsirolimus and vatalanib [14], [47].

Upon reviewing the current literature the biomarkers CD31, pPDGFRα and -β, Ki67, and (p)VEGFR1 have not been demonstrated to be associated with sunitinib response. Instead, associations were observed between response to sunitinib and lower levels of plasma soluble proteins such as sVEGFR3 and VEGFC, higher expression of the soluble isoforms of VEGFA (VEGFA_121_, VEGFA_165_) and normal levels of C-reactive protein [36], [48], [49].

Other biomarkers for TKI activity might be cytokines and angiogenic factors (CAFs) or single nucleotide polymorphisms (SNPs). Zurita *et al.* measured concentrations of 52 plasma CAFs in patients receiving sorafenib alone or with interferon α and identified a CAF signature of six markers (osteopontin, VEGF, CA9, collagen IV, VEGFR2 and tumor necrosis factor-related apoptosis-inducing ligand) associated with PFS benefit from the combination of both therapeutics [50]. Additionally, there are early studies showing associations between SNPs in genes of drug metabolism (CYP3A5), drug transporters (ABCB1) as well as genes of the angiogenic pathway (VEGF, VEGFR2, VEGFR3) and patients’ predisposition for longer survival and response to TKIs [51], [52], [53].

To date, the studies published on the evaluation of markers related to the sunitinib response only used best response for data analyses [14], [20], [21] and therefore, the present study is the first examining molecular markers in regard to different periods of response. Here we showed that some molecular markers (CA9 and CD31) were correlated with an early response, whereas other markers (VEGFR1 and -2) were related to a long-term response. The observed varying importance of these markers for different response periods might be partly explained by the effects of sunitinib on the vascular system and the tumor tissue, which can initiate new VEGF-independent pathways during therapy. For example, patients with higher protein levels of VEGFR1 and -2 in endothelial cells (vessel staining) responded better to the sunitinib treatment than those with lower levels. Patients with a higher VEGFR1 and -2 vessel staining showed a long-term response to sunitinib, because this TKI can probably inhibit vessel-bound receptors more permanently. In contrast, CA9, which is only expressed by tumor cells, might be more important at therapy initiation and possibly looses its importance during the course of treatment due to development of acquired resistance. The mechanisms of resistance to targeted therapy have not been fully understood but the maintenance of protein kinase activation during sunitinib treatment may be involved in the acquisition of a resistant phenotype to sunitinib in a RCC cell line [54]. Furthermore, it has been suggested that the development of resistance is accompanied by reestablishment of vasculature that is less dependent on VEGF. Proteins such as fibroblast growth factor, ephrins and angiopoietin family proteins, interleukin-8 and placental growth factor are thought to be involved in resistance to VEGF therapy [55]. In a new study, Pénzválto *et al.* tested 45 cancer cell lines for sensitivity to different TKIs and showed that the most cross-resistance associated genes were related to sunitinib-resistance [56]. Genes such as LGALS8 (lectin), RAB17 (member RAS oncogene family) and EpCAM (epithelial cell adhesion molecule) showed correlations between expression levels and survival of RCC patients treated with sunitinib and might represent new candidates to identify patients who may benefit from sunitinib therapy.

Univariate and multivariate analyses in the present study included clinicopathological parameters, molecular markers and sunitinib response that correlated either by trend or significantly with PFS and OS. Most notable are the significant associations of CA9, CD34, HIF-1α, PDGFRα, VEGFR1 and -3 as well as a response after 6 and 9 months with PFS and OS. Most of these markers also demonstrated in the multivariate Cox proportional hazards regression models for PFS and OS that they represent prognostic markers independent of pT stage, Fuhrman grading and the combined M/N stage. In previous studies protein expression of CA9, VEGFR1 and -2 as well as PDGFRα and -β were analyzed by uni- and multivariate Cox-regression, of which high CA9 and VEGFR2 expression was significantly associated with longer disease-specific survival and PFS, respectively, in multivariate analyses [21], [33]. Theses results support the hypothesis that expression of molecular markers in tumor specimens might predict prognosis and survival of sunitinib-treated metastatic ccRCC patients.

Following the marker analyses with regard to patients’ response and survival we conducted analyses of VHL mutations as well as copy number alterations to verify their potential effects on VHL target gene expression. According to the literature, approximately 70% of sporadic and 60% of metastatic ccRCC patients showed VHL alterations [57], [58] which is consistent to our results. In the present study the inactivation of the VHL gene by the alterations mentioned above elicited a reduction in VHL and an increase in CA9 protein expression level, whereas HIF-1α and VEGFA levels displayed only low gain in patients with VHL changes. Turner *et al.* detected VHL mutations in 56% of ccRCC patients, of which about 69% expressed HIF-1α [59]. Interestingly, protein expression of HIF-1α might also be independent of the VHL status [60]. In contrast, tumors with VHL mutation exhibited a significantly higher CA9 expression than those without [33]. Furthermore, Patard *et al*. reported longer progression-free and disease-specific survival to be predicted by VHL mutation and high CA9 protein levels. In agreement with our results CA9 was an independent prognostic factor in multivariate analysis [13], [33]. These findings support the hypothesis that CA9 may be a viable biomarker for prediction of sunitinib response. Therefore, external validation of these data in an independent patient cohort is needed to confirm reproducibility and transferability of the results.

Further biomarkers might be possibly used for a blood test that measures the levels of markers associated with a good response and thus for identifying patients with a long-term response. Patients who most likely would not benefit from sunitinib therapy should instead possibly receive other TKIs such as pazopanib, axitinib or mTOR-inhibitors like everolimus as well as temsirolimus, although a clinical benefit of this approach has to be shown by further studies. The multi-tyrosine kinase inhibitor pazopanib is the preferred treatment option next to sunitinib but its tolerability might be better than this of sunitinib and more patients favored pazopanib [61], [62]. Next to everolimus [63], axitinib is the preferred choice in second line treatment because of its clinical activity superior to sorafenib and ongoing trials explore this TKI in first line treatment of metastatic ccRCC [64].

Some limitations of the present study have to be addressed. Our research was limited by the relatively small retrospective series of 42 patients with available follow-up data and tumor tissue. Consequently, subgroups of responses and adverse effects are confined in size, which may explain why we were not able to show significant association between sunitinib response and hypertension, as observed previously [22]. In addition, we investigated a large number of molecular markers to investigate the relevance of the angiogenic pathway as well as related proteins in a small cohort of patients that may cause random associations with response or survival. Since we investigated first-line sunitinib treatment which is usually used as a first step in a sequence of systemic treatments, we used a combined endpoint of disease progression and death to assess the efficacy of initial treatment. This endpoint may be affected by different follow-up regimens and the complexity of response evaluation in sunitinib treatment. Considering OS could be an alternative, which could, however, be biased by different subsequent treatments. Therefore, a validation of the results in a different sample cohort would be desirable.

## Conclusion

A number of markers was associated with clinicopathological parameters, PFS and OS of patients with metastatic ccRCC treated by sunitinib. Increased CD34 MVD, HIF-1α score and VEGFR3 vessel staining showed significant correlations with longer PFS in univariate analyses. Prolonged OS was associated with high CA9 score, intensity and membrane staining, strong VEGFR1 vessel staining and low PDGFRα score. Furthermore, the response after 9 months was significantly associated with longer PFS and OS. Molecular markers such as CA9, CD31, HIF-1α, VEGFR1 and -2, pVEGFR1, pPDGFRα and -β as well as Ki67 might serve as predictive markers for a sunitinib response and avoid dispensable therapies and unnecessary adverse effects. Multivariate analyses identified CA9 and a response after 9 months as independent prognostic factors for OS. Our findings may have important implications for the prediction of patients’ response to sunitinib, but have to be validated in independent studies.

## Supporting Information

Figure S1 **Representative Images of IHC staining in non-malignant and malignant kidney specimens.** Representative images for each marker staining in non-malignant (TF) and malignant (TU) kidney specimens. The cytoplasm staining of positive stained tumor cells was investigated for all markers except for CD31, CD34 and Ki67. A complete enclosing membrane staining of tumor cells was considered as positive CA9 membrane staining. A nuclear staining was observed for HIF-1α, pVEGFR1, Ki67 and SVV. CD31 and CD34 are markers for microvessel density (MVD), which was determined per core area. Vessel staining means the positive staining of markers such as VEGFR1, -2 and -3, PDGFRα and -β in endothelial cells of the vascular system. Exemplary images of stained non-malignant kidney specimens are only shown for comparative reasons. Scale is 50 µm in each image.
(TIFF)

Table S1 **Antibodies.**
(DOC)

Table S2 **Raw data of immunohistochemical staining, mutation and copy number analyses indicated for tumor tissue.**
(XLS)
