# Supplementary figures and images for: Analyses of Potential Predictive Markers and Survival Data for a Response to Sunitinib in Patients with Metastatic Renal Cell Carcinoma
Source: PLoS One. 2013 Sep 27;8(9):e76386. doi: 10.1371/journal.pone.0076386 (PMC3785463; doi:10.1371/journal.pone.0076386)

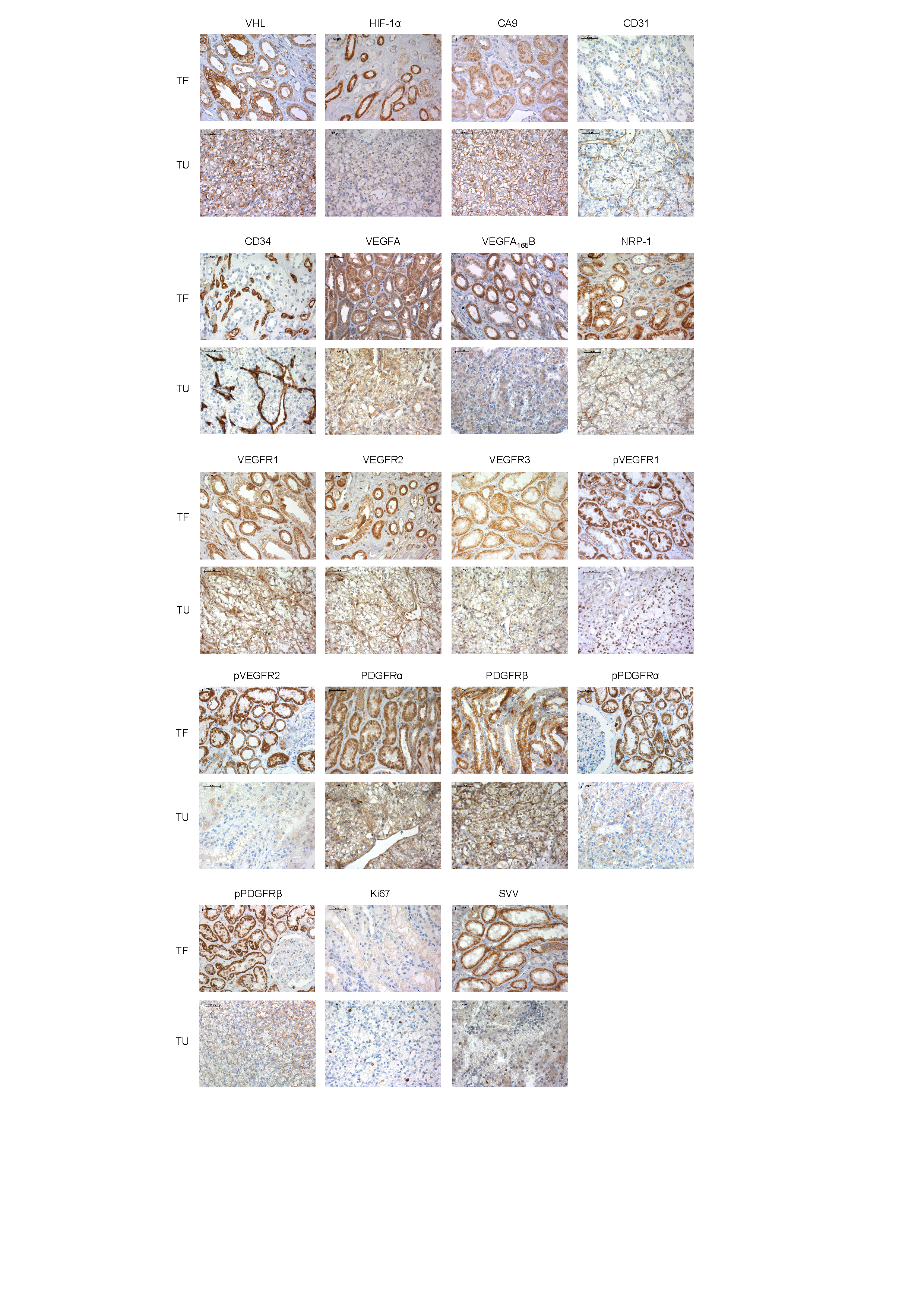

Supplement: Figure S1 — Representative Images of IHC staining in non-malignant and malignant kidney specimens. Representative images for each marker staining in non-malignant (TF) and malignant (TU) kidney specimens. The cytoplasm staining of positive stained tumor cells was investigated for all markers except for CD31, CD34 and Ki67. A complete enclosing membrane staining of tumor cells was considered as positive CA9 membrane staining. A nuclear staining was observed for HIF-1α, pVEGFR1, Ki67 and SVV. CD31 and CD34 are markers for microvessel density (MVD), which was determined per core area. Vessel staining means the positive staining of markers such as VEGFR1, -2 and -3, PDGFRα and -β in endothelial cells of the vascular system. Exemplary images of stained non-malignant kidney specimens are only shown for comparative reasons. Scale is 50 µm in each image. (TIFF) [file pone.0076386.s001.tiff]
