# Supplementary material for: Analyses of Potential Predictive Markers and Survival Data for a Response to Sunitinib in Patients with Metastatic Renal Cell Carcinoma
Source: PLoS One. 2013 Sep 27;8(9):e76386. doi: 10.1371/journal.pone.0076386 (PMC3785463; doi:10.1371/journal.pone.0076386)
Supplement: Table S1 — Antibodies. (DOC) [file pone.0076386.s002.doc]

**Table S1.** Antibodies.

| **Primary antibody** | **Comment** | **Source** | **Antigen retrieval** | **Dilution** |
| --- | --- | --- | --- | --- |
| Monoclonal VHL | Ig32 | BD Biosciences, Heidelberg, Germany | Citrate buffer pH 6.0 | 1/100 |
| Monoclonal HIF-1α | H1alpha67 | Novus Biologicals; Cambridge, UK | Citrate buffer pH 6.0 | 1/100 |
| Monoclonal M75 (CA9)^1^ | - | University Medical Center Nijmegen Experimental Urology | Citrate buffer pH 6.0 | 1/200 |
| Monoclonal CD31 | JC70A | Dako Deutschland GmbH; Hamburg, Germany | Citrate buffer pH 6.0 | 1/5000 |
| Monoclonal CD34 | QBEnd 10 | Dako Deutschland GmbH; Hamburg, Germany | Citrate buffer pH 6.0 | 1/500 |
| Polyclonal Flt-1 (VEGFR1) | C-17 | Santa Cruz Biotechnology; Santa Cruz, USA | Citrate buffer pH 6.0 | 1/150 |
| Monoclonal Flk-1 (VEGFR2) | A-3 | Santa Cruz Biotechnology; Santa Cruz, USA | Citrate buffer pH 6.0 | 1/500 |
| Polyclonal Flt-4 (VEGFR3) | C-20 | Santa Cruz Biotechnology; Santa Cruz, USA | Citrate buffer pH 6.0 | 1/1000 |
| Polyclonal pVEGFR1 | (Tyr 1333) | Abcam; Cambridge, UK | Tris-EDTA pH 8.0 | 1/400 |
| Monoclonal pVEGFR2 | (Tyr 1175) | Cell Signaling Technology, Inc.; Danvers, USA | Tris-EDTA pH 8.0 | 1/900 |
| Monoclonal VEGFA | VG1 | Novus Biologicals; Cambridge, UK | Tris-EDTA pH 9.0 | 1/800 |
| Monoclonal VEGF_165_B | MRVL56/1 | Abcam; Cambridge, UK | Citrate buffer pH 6.0 | 1/1000 |
| Polyclonal NRP-1 | ZMD.223 | Life Technologies GmbH, Darmstadt, Germany | Citrate buffer pH 6.0 | 1/150 |
| Polyclonal PDGFR-α | C-20 | Santa Cruz Biotechnology; Santa Cruz, USA | Tris-EDTA ph 9.0 | 1/300 |
| Polyclonal PDGFR-β | P-20 | Santa Cruz Biotechnology; Santa Cruz, USA | Tris-EDTA pH 9.0 | 1/400 |
| Polyclonal pPDGFR-α | (Tyr 720) | Santa Cruz Biotechnology; Santa Cruz, USA | Tris-EDTA pH 9.0 | 1/550 |
| Polyclonal pPDGFR-β | (Tyr 751)-R | Santa Cruz Biotechnology; Santa Cruz, USA | Tris-EDTA pH 9.0 | 1/1000 |
| Monoclonal Ki-67 | MIB-1 | Dako Deutschland GmbH; Hamburg, Germany | Citrate buffer pH 6.0 | 1/500 |
| Polyclonal SVV | - | Novus Biologicals; Cambridge, UK | Citrate buffer pH 6.0 | 1/2000 |

^1^donation from E. Oosterwijk
